# Validation and Adaptation of the Slovak Version of the Childhood Trauma Questionnaire (CTQ)

**DOI:** 10.3390/ijerph18052440

**Published:** 2021-03-02

**Authors:** Martina Petrikova, Natalia Kascakova, Jana Furstova, Jozef Hasto, Peter Tavel

**Affiliations:** 1 Olomouc University Social Health Institute, Palacky University Olomouc, Univerzitní 22, 77111 Olomouc, Czech Republic; natalia.kascakova@oushi.upol.cz (N.K.); jana.furstova@oushi.upol.cz (J.F.); j.hasto.tn@gmail.com (J.H.); peter.tavel@oushi.upol.cz (P.T.); 2 Psychiatric-Psychotherapeutic Outpatient Clinic, Heydukova 27, 81108 Bratislava, Slovakia; 3 Department of Social Work, St. Elizabeth College of Health and Social Work, Palackeho 1, 81102 Bratislava, Slovakia; 4 Department of Psychiatry, Faculty of Medicine, Slovak Medical University, Limbova 12, 83303 Bratislava, Slovakia

**Keywords:** emotional abuse, physical abuse, sexual abuse, emotional neglect, physical neglect, validation, Childhood Trauma Questionnaire

## Abstract

Background: Previous research has shown a strong relationship between childhood trauma and worsened physical and mental health. The Childhood Trauma Questionnaire (CTQ) is a commonly used tool assessing early traumatic experiences. The aim of this study was to verify the psychometric properties of the Slovak version of the CTQ. Methods: Data were collected on a representative Slovak sample (N = 1018, mean age 46.24 years, 48.7% of men). The dimensional structure of the CTQ was tested by confirmatory factor analysis (CFA); convergent validity was assessed using the Adverse Childhood Questionnaire (ACE-IQ). Results: CFA confirmed the standard 5-factor CTQ model. The subscales of the CTQ and the ACE-IQ questionnaires showed moderate to high correlations. The internal consistency of the scale was found to be acceptable. Emotional neglect (EN) was reported in 48.1%, physical neglect (PN) in 35.8%, emotional abuse in 15.8%, physical abuse (PA) in 11.0%, and sexual abuse (SA) in 9.1% of the Slovak population, according to the scoring, when even low abuse or neglect is assessed as trauma. Conclusion: The CTQ questionnaire fulfilled the validation criteria and appeared to be a suitable method for assessing retrospectively reported childhood trauma experiences in the Slovak population.

## 1. Introduction

The available literature indicates that trauma in childhood and later life is often strongly associated with impaired physical and mental health [1,2,3].

Among the risk factors for mental health are family conflicts, separation from parents during early childhood, neglect, violence, sexual, physical and emotional abuse, unemployment, a large number of family members, parental crime, disharmonious families, parents’ mental illness, parental loss, divorce and bad relationships with peers [4,5]. Adverse childhood experiences affect the nervous, endocrine, and immune systems of both children and adults, which means that they can cause significant biological changes and affect physiological response to adult stress [6]. Childhood trauma experiences pose a higher possibility for the later development of alcoholism, depression, and eating disorders; a higher occurrence of dangerous sexual behavior, HIV and AIDS; an increased risk of heart diseases, cancer, other chronic diseases [7], later overweight, physical inactivity, and smoking; and higher odds of problem alcohol consumption, problem behavior, and the occurrence of mental illnesses [8]. Women with a history of childhood sexual abuse have a higher probability of revictimization [9]. The effects on mental health are significant, too. Several studies have demonstrated the associations between childhood trauma experiences and the odds of mental illness, risky sexual behavior, and substance abuse [1], or the possibility of anxiety disorders [10,11,12], depression in adulthood [11,12,13,14], sleep problems [11], or premenstrual syndrome [15]. Such significant and prolonged stress in childhood can result in impaired health and overall worse well-being [7]. In other studies, associations were found between children’s trauma experiences and increased levels of spirituality [16].

The Childhood Trauma Questionnaire—Short Form (CTQ) is often used to examine reported experiences of child abuse and neglect. The shortened version of the original 70-item questionnaire consists of 28 items (CTQ) [17]. The psychometric properties of the CTQ were verified on a representative and a clinical sample of respondents. The CTQ has been validated in several countries: On representative samples in Germany [18,19], in the Czech Republic [20], in Memphis, USA [21], and on clinical samples in Brazil [22], Spain [23], the Netherlands [24], Germany [25], and Denmark [26]. The CTQ has also been used in many association studies, e.g., to monitor the associations between childhood trauma experiences and chronic pain and anxiety [27], substance use disorder in Taiwanese patients [28], to compare the experiences of trauma in South African women with or without HIV [29], and to support the idea that childhood trauma experiences and adult attachment style are associated with higher use of negative religious coping strategies [30]. The CTQ was also used to examine the prevalence of traumatic experiences in South Korean adults [31].

A meta-analytic study reports the following prevalence of childhood trauma experiences detected retrospectively (worldwide prevalence): 36.3% for emotional abuse; 22.6% for physical abuse; 7.6% and 18% for sexual abuse of boys and girls, respectively; 18.4% for emotional neglect; and 16.3% for physical neglect [32]. The Central Office of Labour, Social Affairs and Family of the Slovak Republic registers cases of ill-treatment in Slovakia. In 2014, 424 children who were bullied, abused, and mistreated in various forms were registered in Slovakia. In 2017, the number increased to 974. The report on the social situation of the Slovak population for 2018 states that the offices have taken action for 1259 children in the cases of domestic violence, abuse, and neglect of children [33]. The Ministry of Labour, Social Affairs and Family of the Slovak Republic registers 124 cases of physical abuse, 28 cases of psychical abuse, 218 cases of sexual abuse, 10 cases of registered bullying, 870 cases of neglect, and 9 cases of the use of children for exploitation. The highest incidence of child abuse was in the age range from 6 to 15 years, for sexual abuse up to 18 years old. However, it is important to realize that these are only registered cases, and the overall prevalence of traumatization may be much higher [34]. In 2013, the Institute for Labour and Family Research (Ministry of Labour, Family and Social Affairs of the Slovak Republic) summarized the results of the prevalence of violence against children in a representative sample of 1560 children aged 14 and 15. Psychological violence was reported in 20.6% of children, physical violence in 23.2%, and sexual violence in 7.1% of children. Neglect involving both emotional and physical abuse was present in 9.4% of surveyed children. One of the questions was: “Do you know a child or children in your neighborhood who have been exposed to any form of violence, physical or psychological abuse, or sexual abuse or child neglect, when he or she does not have adequate care?”, to which 21.7% of respondents answered in the affirmative [35].

It is important to address the topic of childhood traumatization due to its possible adverse effects on health in adulthood. However, for targeted detection and subsequent prevention and resolution of this issue, it is necessary to validate a suitable tool for measuring reported experiences of traumatization in our country retrospectively. The aim of this study is to examine the psychometric properties of the Slovak version of the Childhood Trauma Questionnaire (CTQ) on a representative Slovak sample and to explore the prevalence of retrospectively reported traumatization in adults in various sociodemographic groups of the Slovak population. To assess the convergent validity of the questionnaire, we used the ACE-IQ (Adverse Childhood Experiences International Questionnaire), which also assesses different types of child abuse and neglect [7].

## 2. Methods

### 2.1. Research Sample and Method of Data Collection

Data collection took place in April 2019 through a professional research agency in the form of personal interviews with trained administrators. The sample of respondents was compiled on the basis of data from the Statistical Office of the Slovak Republic on the structure of the adult population in terms of gender, age, education, nationality, size of place of living, and region of living. The research sample was a representative sample of the Slovak adult population, consisting of 1018 participants, 496 men and 522 women, aged 18–85 years (average age 46.2). The descriptive characteristics of the research sample are listed in Table 1.

Respondents’ answers were collected by the method of computer-assisted personal interviewing (CAPI). CAPI is a method of interviewing that significantly saves time and eliminates errors in recording answers [36].

### 2.2. Measures

#### 2.2.1. Childhood Trauma

The original CTQ scale was developed by Bernstein and Fink [37], who defined 5 different types of childhood abuse and neglect. Emotional abuse (EA) was perceived as a verbal attack that disrupted a child’s self-esteem and was degrading or directed against the child. Physical attacks on a child that posed a chance of or caused injuries were defined as physical abuse (PA). Sexual abuse (SA) was defined as sexual contact or intercourse of a person who was usually in a superior position to the child and was at least 5 years older than the victim. Failure of a parent or a caregiver to meet child’s emotional and psychological needs, love, upbringing, and support was defined as emotional neglect (EN). Physical neglect (PN) was characterized as a failure of a parent or a caregiver to provide the child’s basic needs (food, clothing, safety, shelter, health care) [37].

Each subscale of the CTQ was answered by 5 questions from the questionnaire, which were rated on a scale from 1 (never) to 5 (very often). The minimum score achieved in each subscale was 5; the maximum was 25. The questionnaire also allowed the severity of abuse and neglect to be categorized at 4 levels: (1) None to minimal, (2) low to medium, (3) moderate to severe, and (4) severe to extreme [17]. The cut-off limits were different for each type of trauma: Emotional abuse 9, physical abuse 8, sexual 6, emotional neglect 10, physical neglect 8 [37]. The clinically derived cut-off scores, based on a dichotomous differentiated score, were 10 for EA, 8 for PA, 8 for SA, 15 for EN, and 8 for PN [38]. Walker et al. derived dichotomous differentiated scores using cut-off limits with very good sensitivity and specificity (≥0.85) by using a structured clinical interview focusing on the history of clinically significant abuse and neglect. These cut-off limits generally identified women who had moderate to severe levels of abuse or neglect [38].

The MD-scale (Minimization and Denial Scale) is a part of the CTQ and it consisted of the 3 questions not classified in the abuse and neglect subscales, was used to reveal the denial of problems in childhood. However, in our study, for capacity reasons, we did not process the MD-scale.

#### 2.2.2. Adverse Childhood Experiences

The Adverse Childhood Experiences International Questionnaire (ACE-IQ) [7] was used to assess the convergent validity of the CTQ. The ACE-IQ contained 31 questions and monitors 13 types of adverse childhood experiences: Physical and emotional abuse, sexual abuse, alcohol abuse problems in the family, caring for a family member who is physically or mentally ill, domestic violence, divorce or parental separation, emotional and physical neglect, alcohol and substance abuse in the family, collective violence and bullying [39].

The CTQ and the ACE-IQ were parts of a broader questionnaire battery measuring a variety of concepts, but for the purpose of the current study, we focused just on those variables. The Slovak versions of the CTQ and the ACE-IQ were obtained by a back-translation procedure: The original questionnaires were translated from English by 2 freelance translators—trauma experts—then back into English and finally the translations were corrected. A pretest of the questionnaires was used on a sample of 10 respondents of both genders, various ages, and educational categories. After the pretesting, minor modifications were made, especially in the area of unclear instructions, in the presence of the experts in trauma and psychological and sociological research.

### 2.3. Statistical Analyses

All the statistical analyses were performed using the IBM SPSS Statistics software version 21 (IBM Corp., Armonk, New York, NY, USA) and the R software, version 3.6.0 (R Foundation for Statistical Computing, Vienna, Austria). Normal distribution of the data was verified using histograms and the Shapiro–Wilk test. The data did not meet the assumption of normality; therefore, non-parametric statistical methods were used for the analyses. The factor structure of the CTQ scale was assessed using Confirmatory Factor Analysis (CFA). The DWLS (Diagonally Weighted Least Squares,) method based on the matrix of polychoric correlations was used to estimate the CFA parameters. The CFA was calculated using the Lavaan package [40]. Several measures were examined as fitting parameters of the CFA models: The Comparative Fix Index (CFI), the Tucker-Lewis Index (TLI), Root Mean Square Error of Approximation (RMSEA), and Standardized Root Mean Square Residual (SRMR). The internal consistency of the CTQ scale was evaluated with the Cronbach’s alpha and McDonald omega coefficients. Convergent validity was assessed using the nonparametric Spearman correlation between the CTQ subscales and selected questions of the ACE-IQ scale. To compare the sociodemographic groups, nonparametric tests were used: The Mann–Whitney U test for comparison of 2 groups and the Kruskal–Wallis test with Dunn–Bonferroni correction for multiple group comparison. The significance level was set at *p* < 0.05 for all statistical significance testing. In addition to the p-values, the Cohen’s d (in the text “d”) and partial η^2^ (eta squared, in the text “e”) effect size coefficients were evaluated.

## 3. Results

### 3.1. Psychometric Properties of the CTQ Questionnaire

As a first step, the statistics of the CTQ items, including the item-total correlation coefficients, were evaluated (see Table 2).

### 3.2. Factor Structure of the CTQ Scale

**Figure 1:**
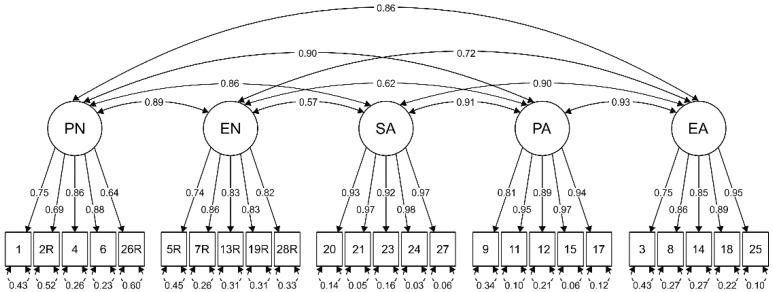
Structural equation modeling (SEM) path diagram of the CTQ scale with items distributed into five standard subscales.

Due to the ordinal nature of the data, confirmatory factor analysis (CFA) based on the polychoric correlation matrix was used for validation of the factor structure. We considered a standard five-factor model of the CTQ scale based on the theoretical background of the scale [17]. The five-factor model distributes 25 items into factors and leaves three items (10, 16, and 22) unclassified. The fit parameters of this model were acceptable: χ^2^ (265) = 1088.5, *p* < 0.001, χ^2^/df = 4.1, CFI = 0.996, TLI = 0.995, RMSEA = 0.055 (90% CI: 0.052–0.058), and SRMR = 0.058. The loadings of all the items were moderate to high, with values ≥0.63 (see Figure 1).

### 3.3. Reliability

The internal consistency of the CTQ was verified for the whole 28-item scale. The analysis showed acceptable reliability of the scale, with Cronbach’s alpha = 0.84 (95% CI: 0.82–0.85). The alpha value slightly decreased after removing individual items from the scale, except for items 10, 16, and 22. Those items formed the MD-scale (Minimisation and Denial Scale) and were not included in any of the five subscales of the CTQ. Excluding these three items from the scale increased Cronbach’s alpha to 0.92 (95% CI: 0.91–0.93). We also verified the reliability of the scale using the McDonald’s omega coefficient, which was more suitable for multidimensional scales. For the complete CTQ scale (with 28 items), the hierarchical omega coefficient reached a value of 0.76, and the total omega reached 0.96. After excluding unclassified items 10, 16, and 22, the hierarchical omega value increased slightly to 0.80, while the value of the total omega remained unchanged. These values indicate that the reliability of the CTQ scale in the Slovak environment was high. The internal consistency of the CTQ also appears to be acceptable for the subscales. The lowest reliability was acquired by the PN subscale. The reliability values of the individual subscales are given in Table 3.

### 3.4. Convergent Validity

In the next step, we assessed the correlations of the CTQ questionnaire subscales with the subscales of the ACE-IQ questionnaire, which explores the reported trauma from childhood. The individual subscales of the questionnaires are moderately correlated (see Table 4).

### 3.5. The Occurrence of Traumatization and Sociodemographic Differences

Table 5 shows the percentage of occurrence of individual types of traumatization. According to the scoring from Bernstein and Fink [37], where even a low score of abuse or neglect was considered to be a trauma, 58.6% of respondents experienced traumatization, while 41.3% of respondents experienced trauma according to the clinically derived scoring from Walker et al. [38].

Table 6 shows the characteristics of the data set and the results of a nonparametric comparison of the summary score of the CTQ subscales (Mann–Whitney U test and Kruskal–Wallis test). The *p*-value corresponds to a comparison of all groups, while the relationships in parentheses are the result of multiple group comparisons.

The results of nonparametric analyses showed differences in the prevalence of reported trauma in different sociodemographic groups. A significant difference was found between males and females in the PA and PN subscales. Women had lower mean values in both the PN and PA subscales compared to men, but only with a low effect size (Cohen’s d = 0.12–0.13; e = 0.003–0.004). The results indicated that the oldest group of participants (65 years and older) reached the highest mean values in the PA, EN, and PN subscales. In the PA and PN subscales, seniors achieve significantly higher values than all other age groups up to 55 years (with effect size d = 0.29–0.35; e = 0.021–0.029). The group of respondents living with a partner without marriage had the highest mean values in the EA, PA, EN, and PN subscales. In contrast, the lowest values were reached by respondents living with their parents (with the effect size d = 0.19–0.34; e = 0.009–0.028). Participants with a university degree reported significantly lower EA, PA, EN, and PN values than respondents with primary education or a completed apprenticeship. Respondents with a completed secondary education had significantly lower values in the EA and PA subscale than respondents with a lower education level (with effect size d = 0.25–0.28; e = 0.015–0.019).

## 4. Discussion

### 4.1. Psychometric Properties of the Slovak Version of the CTQ

The correlations between the subscales of the questionnaire in our study were moderate to strong, similar to several studies [17,19,20,22,26,41]. The results suggest that the strongest correlations occurred between the EA and PA subscales and the EN and PN subscales. The results of confirmatory factor analysis (CFA) indicate medium to high loadings of all items, similarly as in a Danish clinical trial study [26] and a Taiwanese sample of respondents with substance use disorders [28]. Acceptable loadings of the items were also confirmed during validation on a German [18] and a Czech representative sample [20].

The internal consistency of the EN, SA, PA, and EA subscales appear to be high. The PN subscale has the lowest values for both Cronbach’s α and McDonald’s ω, which is in line with several validation studies [14,18,20,22]. The lower values of the internal consistency of the PN subscale can be explained by the diversity of items in this subscale [20]. The PN subscale contains items focused on poverty (enough food, clothes) as well as items focused on a lack of care (e.g., I knew there was someone who would take care of me and protect me), which besides physical neglect can also represent a lack of emotional care. We can assume that the lower internal consistency of the PN scale is not related to the local Slovak version of the questionnaire but probably reflects a problem of the original heterogenous design of the physical neglect subscale.

The results of convergent validity indicate moderate correlations between the CTQ and ACE-IQ subscales. In the present study, the strongest correlation was found in the SA subscales, and the lowest value of the correlation coefficient was reached by the EN and PN subscales. A medium-to-strong positive correlation for EA, PA, SA, and PN between the questionnaires was also confirmed by the results on a specific sample of 253 Nigerian prisoners (220 men) [42] and by a validation study on 77 pregnant American women [43].

### 4.2. The Occurrence of Traumatization and Sociodemographic Differences

The occurrence of three or more types of traumatization, according to scoring from Bernstein and Fink [37], was reported in 14.7% of 1018 respondents. In comparison, in a German sample of 2504 respondents, the occurrence of three or more types of traumatization reached 16.6% [19], and in Czech research on a sample of 1800 participants, 17.4% [20].

In the present study, the absence of traumatization was reported in over 40% of respondents according to the Bernstein and Fink scoring [37] and in almost 60% of respondents according to clinically derived cut-off scores [38]. In a German study on a representative sample, almost a third of participants had no trauma experience [19]. Another German study [44] states that out of a total of 2487 respondents, 31% of people reported at least one experience of child abuse.

In our sample, the most common types of traumatization were PN and EN. A high occurrence of EN was also found in studies on representative samples in the Czech Republic [20] and in Germany [19,44]. In the German representative sample [19], PN was even more common than in the Slovak sample. We assume the reason may be a stronger impact of the post-war period on Germany and a greater exposure of the population to poverty and difficult living conditions. A study from 2014 examined the effect of World War II on people in European countries, including Germany [45]. Their findings point to the fact that lower education, less life satisfaction, and poor health are related to the effect of hunger, dispossession, persecution, and the absence of a father during the war.

The lowest occurrence of traumatization was found in the SA subscale. Sexual abuse as the lowest reported trauma was also found in a German validation study [14]. In another German study [19] with 2504 respondents, the prevalence of SA was up to 12.6%. The lower prevalence of SA in our sample compared to the German sample could be explained as a consequence of the continuing taboo and stigma in our country. Fear and reluctance to talk openly about experiences of sexual abuse reduce the number of registered cases, and we assume that the actual percentage of SA in Slovakia is much higher.

The results of several studies explain that only a few children confide the experience of SA shortly after the incident. One-third of the victims would share this experience during their childhood. Another third would confide within an interval of 8–15 years after the experience. The last third of the victims never confide the experience of SA, even in adulthood, or only in exceptional cases. There are several reasons why victims do not share their experience of sexual abuse: Fear of the perpetrator, inability to identify the situation (children do not know that what they went through was wrong), inappropriate reactions to the social environment (fear that they would not be trusted), etc. [46].

The results of statistical analyses suggest differences in the traumatization in different sociodemographic groups, similarly to the German [14,44] and Czech studies [20]. In our research sample, women achieved lower average values in the PN subscales compared to men, similarly to the Czech research sample [20]. Women also had lower scores in PA, similar to the German study [14]. In addition, in a South Korean study, males reported more childhood trauma experiences than females [31]. We assume that this may be related to differences in upbringing and more frequent use of physical punishment against boys compared to girls and may depend on individual and cultural factors. This phenomenon was also confirmed by a Swiss study [47], which states that boys are physically punished more often than girls. Other studies [14,44] further describe that women reported more frequent SA experiences than men. This phenomenon was not confirmed in our sample, and there is no statistically significant difference between men and women in the SA subscale.

Respondents over the age of 65 achieved higher average values in the PA, EN, and PN subscales compared to other groups. A higher proportion of reporting those types of abuse and neglect in people older than 50 years was also confirmed in other studies [14,20,44]. This phenomenon can be explained by the fact that physical punishment was perceived in past decades more as an upbringing tool and was more common [14]. The effect of age on the occurrence of PN proved to be significant because respondents aged 60 and over were exposed to worse conditions during the years when they were growing up in the post-war period. Thus, there is a need for a sensitive interpretation of this subscale with regard to age. Seniors in the Czech study differed in the PN scale mainly compared to the youngest group, which the authors explain by the increase in living standards over the decades and lower living standards in the post-war and communist periods [20]. In the German [14] and Czech [20] studies, older respondents had a lower occurrence of EA compared to younger groups. In our sample, this phenomenon was not confirmed, and the differences between the groups were not statistically significant.

In terms of marital status, the highest mean values in the scales of EA, EN, PA, and PN were achieved by respondents living with a partner without a marriage. The lowest values in these four subscales were reached by participants living with their parents. Other studies also confirmed a higher occurrence of traumatic childhood experiences in respondents who do not live in a marriage [31,38]. The authors explain that living in a close relationship is potentially very intimidating for people who suffered traumatic childhood experiences.

In agreement with several studies [19,20,31,38,44,47], our results also indicate that a higher occurrence of traumatization was demonstrated in respondents with a lower level of education. The results confirm the findings that trauma in childhood affects an individual’s learning abilities and cognitive development [48,49].

### 4.3. Strengths and Limitations

The strength of this study is that it is based on a representative sample. The completed questionnaires had no missing values. However, this study has several limitations. First, the CTQ and the ACE-IQ were parts of a broader questionnaire battery measuring a variety of concepts, and they were placed approximately in the middle of the whole battery. The whole interview with one participant lasted approximately 45–60 min. The standardized interview by the CAPI method was relatively time consuming, and the respondents’ answers could be influenced by increased fatigue and reduced attention. A second limitation is that the history of childhood trauma was based on retrospective reporting, which can be potentially biased. Studies about the validity of retrospective reports of childhood trauma show a tendency to underreport it [50]; therefore, it can be assumed that the real prevalence was much higher.

## 5. Conclusions

The Slovak version of the CTQ questionnaire fulfilled the validation criteria and appeared to be a suitable method for assessing retrospectively reported childhood trauma experiences in the Slovak population. Obtaining a quality tool for recognizing traumatization in childhood and information about the prevalence of traumatization in a Slovak representative sample is important for further examination of associations among traumatization and the formation of relationships and health in adulthood. Data from a representative sample are also a good basis for establishing preventive programs with the goal of reducing the global prevalence of childhood traumatization.

## Figures and Tables

**Table 1:** Description of the research sample.

	N	%
Total	1018	100
**Gender**		
Men	496	48.7
Women	522	51.3
**Age**		
18–24 y	110	10.8
25–34 y	187	18.4
35–44 y	199	19.5
45–54 y	166	16.3
55–64 y	168	16.5
65 y. and more	188	18.5
**Marital status**		
Married	551	54.1
With partner	120	11.8
Single	162	15.9
With parents/siblings	185	18.2
**Education level**		
Primary school	137	13.5
Completed apprenticeship	272	26.7
Secondary school, graduated	382	37.5
University	227	22.3

**Table 2:** The item statistics of the Childhood Trauma Questionnaire (CTQ) scale (*n* = 1018).

CTQ Subscale	Item Number	Mean	Sd	Item-Total Correlation
Physical neglect (PN)	1	1.3	0.70	0.53
	2R	1.7	1.10	0.48
	4	1.2	0.65	0.60
	6	1.2	0.66	0.64
	26R	1.9	1.32	0.42
Emotional neglect (EN)	5R	2.1	1.19	0.49
	7R	1.8	1.05	0.60
	13R	2.1	1.11	0.58
	19R	2.1	1.14	0.56
	28R	1.9	1.16	0.56
Sexual abuse (SA)	20	1.1	0.50	0.60
	21	1.1	0.50	0.62
	23	1.1	0.48	0.56
	24	1.1	0.44	0.64
	27	1.1	0.46	0.61
Physical abuse (PA)	9	1.2	0.65	0.45
	11	1.2	0.57	0.69
	12	1.3	0.64	0.65
	15	1.2	0.56	0.72
	17	1.1	0.49	0.66
Emotional abuse (EA)	3	1.6	0.90	0.55
	8	1.3	0.74	0.61
	14	1.4	0.79	0.65
	18	1.2	0.63	0.65
	25	1.2	0.52	0.68

**Table 3:** Internal consistency of the individual subscales of the CTQ.

	Cronbach α (95 % CI)	McDonald ω
PN	0.64 (0.60–0.67)	0.78
EN	0.85 (0.83–0.86)	0.87
SA	0.94 (0.94–0.95)	0.96
PA	0.88 (0.87–0.89)	0.91
EA	0.84 (0.82–0.85)	0.87

Note: CI = confidence interval.

**Table 4:** Spearman nonparametric correlation coefficients between the subscales of the CTQ and the Adverse Childhood Questionnaire International Questionnaire (ACE-IQ) (*n* = 1018).

	ACE-IQ				
	EA	PA	SA	EN	PN
CTQ					
Emotional abuse (EA)	0.465 ***				
Physical abuse (PA)		0.441 ***			
Sexual abuse (SA)			0.449 ***		
Emotional neglect (EN)				0.417 ***	
Physical neglect (PN)					0.417 ***

Note: *** *p* < 0.001.

**Table 5:** The prevalence of traumatization according to the CTQ.

Occurrence in %	According to Clinical Severity from Walker et al. [38]	According to Bernstein and Fink [37]
Type of traumatization		
Emotional abuse (EA)	11.7	15.8
Physical abuse (PA)	11.0	11.0
Sexual abuse (SA)	6.7	9.1
Emotional neglect (EN)	17.1	48.1
Physical neglect (PN)	35.8	35.8
Number of types of traumatization		
No type of traumatization	58.7	41.4
Traumatization	41.3	58.6
1 type of traumatization	21.3	25.5
2 types of traumatization	9.0	18.4
≥3 types of traumatization	10.9	14.7

**Table 6:** Descriptive characteristics of the data set and nonparametric comparison of the summary scores in individual CTQ subscales in different population groups.

	EA	PA	SA	EN	PN
	M (SD)	M (SD)	M (SD)	M (SD)	M (SD)
Gender					
1. Male	6.66 (2.67)	5.95 (2.35)	5.51 (2.00)	10.16 (4.51)	7.57 (2.96)
2. Female	6.64 (2.99)	5.84 (2.44)	5.61 (2.29)	9.89 (4.40)	7.28 (2.97)
Statistical significance	NS	*p* = 0.025	NS	NS	*p* = 0.035
Age categories					
1. 18–24 years	6.50 (2.76)	5.79 (2.43)	5.50 (1.78)	9.70 (4.35)	6.66 (2.64)
2. 25–34 years	6.65 (3.01)	5.70 (2.29)	5.56 (2.18)	9.71 (4.67)	7.34 (3.01)
3. 35–44 years	6.57 (2.98)	5.97 (2.81)	5.66 (2.46)	9.67 (4.59)	7.29 (3.06)
4. 45–54 years	6.68 (2.72)	5.81 (2.26)	5.65 (2.46)	10.11 (4.33)	7.20 (2.88)
5. 55–64 years	6.68 (2.90)	5.88 (2.27)	5.53 (2.12)	10.21 (4.43)	7.52 (3.01)
6. 65 years and older	6.77 (2.61)	6.15 (2.25)	5.46 (1.78)	10.65 (4.25)	8.20 (2.92)
Statistical significance	NS	*p* < 0.001 (1–6 **, 2–6 ***, 3–6 **, 4–6 *)	NS	*p* = 0.042	*p* < 0.001 (1–5 *, 1–6 ***, 2–6 **, 3–6 ***, 4–6 **)
Marital status					
1. Married	6.54 (2.83)	5.89 (2.41)	5.55 (2.21)	9.80 (4.43)	7.32 (2.89)
2. With partner	7.32 (3.28)	6.17 (2.87)	5.93 (2.80)	11.19 (4.27)	8.33 (3.35)
3. Single	6.72 (2.57)	5.96 (2.16)	5.52 (1.85)	10.40 (4.54)	7.86 (2.95)
4. With parents	6.49 (2.73)	5.65 (2.23)	5.39 (1.70)	9.62 (4.45)	6.75 (2.77)
Statistical significance	*p* = 0.008 (1–2 *, 2–4 *)	*p* = 0.007 (2–4 *, 3–4 **)	NS	*p* = 0.001 (1–2 **, 2–4 **)	*p* < 0.001 (1–2 *, 1–4 **, 2–4 ***, 3–4 ***)
Education level					
1. Primary school	7.18 (3.01)	6.14 (2.36)	5.77 (2.39)	10.90 (4.60)	8.22 (3.40)
2. Completed apprenticeship	6.85 (2.93)	6.07 (2.65)	5.60 (2.15)	10.47 (4.40)	7.57 (2.85)
3. Secondary school, graduated	6.64 (2.98)	5.88 (2.51)	5.66 (2.43)	9.85 (4.44)	7.35 (2.99)
4. University	6.11 (2.25)	5.55 (1.81)	5.24 (1.33)	9.26 (4.34)	6.89 (2.69)
Statistical significance	*p* < 0.001 (1–3 *, 1–4 ***, 2–4 **)	*p* < 0.001 (1–3 *, 1–4 **, 2–3 *, 2–4 **)	NS	*p* < 0.001 (1–4 **, 2–4 **)	*p* < 0.001 (1–3 *, 1–4 ***, 2–4 **)

Note: EA = Emotional abuse, PA = Physical abuse, SA = Sexual abuse, EN = Emotional neglect, PN = Physical neglect; M = Mean, SD = Standard deviation; * *p* < 0.05, ** *p* < 0.01, *** *p* < 0.001; NS = Non-significant.

## Data Availability

Not applicable.
